# Relative bioavailability of selenium yeast, selenomethionine, hydroxyl-selenomethionine and nano-selenium for broilers

**DOI:** 10.3389/fvets.2024.1542557

**Published:** 2025-01-17

**Authors:** Guoqing Liu, Sumei Cao, Liang Huang, Xuanxu Lin, Zheng Sun, Gang Lin, Liyang Zhang, Lin Lu, Xugang Luo, Xiudong Liao

**Affiliations:** ^1^Mineral Nutrition Research Division, State Key Laboratory of Animal Nutrition and Feeding, Institute of Animal Science, Chinese Academy of Agricultural Sciences, Beijing, China; ^2^Department of Animal Science, Hebei Normal University of Science and Technology, Qinhuangdao, China; ^3^Laboratory of Quality and Safety Risk Assessment for Animal Products on Feed Hazards (Beijing) of the Ministry of Agriculture and Rural Affairs, Institute of Feed Research, Chinese Academy of Agricultural Sciences, Beijing, China; ^4^Poultry Mineral Nutrition Laboratory, College of Animal Science and Technology, Yangzhou University, Yangzhou, China

**Keywords:** bioavailability, broiler, selenium concentration, selenium source, selenoprotein expression

## Abstract

Selenium (Se) is an essential trace element for humans and animals. Development and application of new forms of Se sources with lower toxicity and higher bioavailability has been attracting more attention. However, the bioavailabilities of Se from several new Se sources for broilers remain unclear. Therefore, the aim of this study was to assess the relative bioavailabilities of Se from Se yeast (SY), selenomethionine (SM), hydroxyl-selenomethionine (SO) and nano-Se (NS) relative to sodium selenite (SS) for broilers fed a conventional corn-soybean meal diet. A total of 576 one-day-old Arbor Acres commercial male broilers were randomly assigned to 16 treatments with 6 replicate cages per treatment in a completely randomized design involving a 5 (Se sources: SY, SM, SO, NS and SS) × 3 (added Se levels: 0.15, 0.30 and 0.45 mg Se/kg) factorial design of treatments plus 1 (a Se-unsupplemented control) for 21 d. The relative bioavailabilities of Se sources were estimated based on plasma or tissue Se concentrations as well as selenoprotein mRNA expressions and activities in broilers. The results showed that the Se concentrations and glutathione peroxidase (GPX) activities in plasma, liver, breast muscle, pancreas and kidney as well as Se concentration in erythrocytes of broilers, and *Gpx1* and *Selenop* mRNA expressions in pancreas increased linearly (*p* < 0.03) as added Se level increased. Furthermore, the differences (*p* < 0.05) among different Se sources were detected for the Se concentrations in liver, breast muscle, pancreas and erythrocytes, GPX activities in pancreas and kidney. Based on slope ratios from the multiple linear regressions of the above indices, the Se bioavailabilities of SY, SM, SO, NS relative to SS (100%) were 78 to 367%, 67.8 to 471%, 57 to 372%, and 45 to 92%, respectively. The results from this study indicated that the Se from SM, SY and SO are more available to broilers than the Se from SS in enhancing the Se concentrations in liver, breast muscle, pancreas and erythrocytes and GPX activity in pancreas, and the Se from SM had the highest while the Se from NS had the lowest relative bioavailability.

## 1 Introduction

Selenium (Se) is an essential trace element with important functions in human and animal health (1). This micronutrient supports various important cellular and organismal functions, such as antioxidant defense, immunity, reproduction and antitumorigenesis, etc. (2–4). There are more than 40 countries described as having areas with very low soil Se content and most of the soils in China are also marginal to deficient in Se (5, 6). Thus it is common practice to supplement Se because of the diets low in Se (7). Particularly, broiler chicks are very susceptible to dietary Se deficiency due to their fast growth rate (8). On the other side, Se toxicity should not be ignored, which presents a very narrow range between deficient, essential and toxic doses (9). It has been attracting more attention in the development and application of new forms of Se sources with lower toxicity and greater bioavailability (10–14). The Se is considered to be closely related to its chemical form in terms of toxicity, bioavailability, metabolic mechanisms, and physiological roles (15). The inorganic sodium selenite (SS) and organic Se yeast (SY) are commonly used Se in the animal feed industry. Additionally, a few new forms of Se sources, such as organic selenomethionine (SM) and hydroxyl-selenomethionine (SO), and new type of nano-Se (NS), are also currently being used or developed as feed additives. Many studies have demonstrated that the organic Se sources are less toxic and more effective than inorganic SS in increasing tissue Se concentrations, antioxidant ability and immune function in broiler chickens (16–19). Besides, it has been reported that NS have a higher efficiency in promoting selenoenzymes and exhibit less toxicity than SS (8, 20). However, to our knowledge, information is lacking on the bioavailabilities of these new forms of Se sources, therefore, it is necessary to evaluate their relative bioavailabilities for broilers.

The Se metabolic functions have been attributed mainly to its presence in selenoproteins as a form of selenocysteine (21). There are 25 selenoproteins that are recognized in humans, 24 confirmed selenoproteins in chickens, including glutathione peroxidase (GPX), iodothyronine deiodinase (DIO) and thioredoxin reductase (TXNRD) families, selenoprotein P (Selenop), selenoprotein U (Selenou), etc. (22, 23). Nevertheless, few studies have been conducted to determine the bioavailabilities of different Se sources for broiler chickens by using these selenoprotein expressions as biomarkers (24). In general, only tissue Se enrichment indicators are used in several broiler studies (12, 13). Recently, the study from our laboratory have indicated that the GPX and DIO activities in plasma and tissues as well as *Gpx1*, *Gpx4*, *Selenop* and *Selenou* mRNA expressions in various tissues of chickens increased linearly as added Se level increased (25), suggesting these indices might be used to estimate the Se bioavailability. Furthermore, Liu et al. (16) reported that the GPX activity and mRNA expression in pancreas of broilers were sensitive indices for evaluating relative bioavailability of SY, and the Se bioavailabilities of SY relative to SS (100%) were 173 to 306% based on the GPX activity and mRNA expression. But it is still unknown whether the enzyme activity and mRNA expression of selenoproteins in plasma or tissues of broilers could be used to assess the relative bioavailability for the above new forms of Se sources. Therefore, this experiment was conducted to estimate the bioavailabilities of SY, SM, SO, NS relative to SS based on enzyme activity as well as mRNA expressions of selenoproteins, and Se concentrations in plasma, erythrocytes and various tissues of broilers fed a conventional corn-soybean meal diet.

## 2 Materials and methods

All experimental procedures were approved by the Animal Management Committee (in charge of animal welfare issue) of the Institute of Animal Science, Chinese Academy of Agricultural Sciences (IAS-CAAS, Beijing, China), and performed in accordance with the guidelines. Ethical approval on animal survival was given by the animal ethics committee of IAS-CAAS (IAS2021-83).

### 2.1 Experimental design and treatments

A completely randomized design involving in a 5 (Se sources) × 3 (added Se levels) factorial arrangement of treatments plus 1 (an Se-unsupplemented control) were used in this experiment. The 5 Se sources were SY, SM, SO, NS and SS, and 3 added Se levels were 0.15, 0.30 and 0.45 mg Se/kg diet, respectively. These Se sources and Se levels could reflect the actual situation of broiler production. Therefore, there were a total of 16 treatments including the 1 Se-unsupplemented basal diet control.

### 2.2 Animals and diets

A total of 576 one-day-old Arbor Acres male broilers (Huadu Broiler Breeding Corp., Luanping, China) with similar body weight were randomly assigned to each treatment group according to the above experimental design. The 6 chicks in one cage served as one replicate, and each treatment had 6 replicate cages. The chicks were housed in electrically heated, thermostatically controlled cages equipped with fiberglass feeders and water supply in an environmentally controlled room. Environmental temperature was maintained at 35°C for the first 3 d, after which it was gradually reduced by 3°C per week until it reached 24°C. Temperature was then maintained at 24°C until the end of the experiment. Feed and tap water were provided *ad libitum*. The whole experiment lasted for 21 d. Mortality was recorded daily, at 21 d of age, chick weight and feed intake per cage were measured to calculate average daily gain (ADG), average daily feed intake (ADFI), and feed conversion ratio (FCR) from 1 to 21 d of age.

The basal corn-soybean meal diet (Supplementary Table 1) was formulated to meet or exceed the requirements of broilers for all other nutrients except for Se recommended by the Feeding standard of chicken (26). These Se sources were supplied by different special company, respectively. The SS was reagent grade (≥98% purity) and diluted with corn starch into SS premix containing Se 0.1061%. The Se concentration from SY was determined to be 0.2283%. The SM (≥98.5% purity) was diluted with corn starch and contained Se 0.1021%. The SO was also diluted with corn starch and the Se concentration was 0.1066%. The Se concentration of NS was determined to be 0.4654%, and the particle size is 30–60 nm. The above Se sources were added to the basal diet according to the above experimental treatments, and the analyzed Se concentrations of diets are listed in Supplementary Table 2.

### 2.3 Sample collections and preparations

At 21 d of age, 3 birds from each replicate cage were chosen based on the average body weight. Blood samples were obtained through heart puncture and then centrifuged at 3000 × g for 10 min to separate plasma and erythrocyte, and they were frozen at −20°C for analyses of Se concentrations and plasma GPX and DIO activities. Then the selected birds were killed by cervical dislocation for collecting liver, breast muscle, pancreas and kidney samples. A part of samples were stored at −20°C for analyses of Se concentrations, GPX and DIO activities, and another part of samples were stored in liquid nitrogen and then frozen at −80°C for determinations of *Gpx1*, *Gpx4*, *Dio1*, *Selenop* and *Selenou* mRNA expressions. Samples of 3 chicks in one replicate cage were mixed into one sample in the same proportion before testing.

### 2.4 Se concentrations and enzyme activity

The Se concentrations in the feed ingredients, diets, plasma, erythrocyte and tissues were measured using the fluorescence method with a Hitachi 850 fluorescence spectrophotometer (16). The standard reference material bovine liver powder (GBW (E) 080193, National Institute of Standards and Technology, Beijing, China) was used for validation of the Se analysis. The GPX and DIO activities were determined using corresponding commercial assay kits (Nanjing Jiancheng Bioengineering Institute, Nanjing, China; Shanghai Jianglaibio Company Ltd., Shanghai, China) through the colorimetric method, respectively. All of these procedures were conducted in accordance with the manufacturers’ instructions.

### 2.5 RNA extraction, reverse transcription, and real-time quantitative PCR

Total RNA in the liver, breast muscle, pancreas and kidney was extracted by using Trizol Reagent (life technologies, Carlsbad, CA, United States) in accordance with the manufacture’s protocol. The reverse transcription and real-time PCR reactions were performed according to our previous study (16). Primer sequences for *Gpx1*, *Gpx4*, *Dio1*, *Selenop* and *Selenou*, *GAPDH* and *β-actin* (Supplementary Table 3) were used for amplification reactions according to their gene sequences published in GenBank, respectively. The 2^–ΔΔCT^ method (27) was used to calculate the mRNA expression of the target gene, and was normalized by the geometric mean of *β*-actin and *GAPDH*.

### 2.6 Statistical analyses

The single degree of freedom method was used to test the significance of the differences between all Se-supplemented groups and the control group (28, 29). Data excluding the control were analyzed by two-way ANOVA using the general linear model procedure of the SAS 9.4 (SAS Institute Inc., Cary, NC, USA). The model included the effects of Se source, Se level, and their interaction. The cage was the experimental unit. Orthogonal polynomials were used to assess linear and quadratic responses to added Se level. Relative bioavailability values were determined using SS as the standard source by slope ratio comparisons from multiple linear regressions (30). The regressions were calculated using daily dietary Se intake as the independent variable (16, 31). Slope ratios and their SE were estimated using the method of error propagation (32). Differences among the Se sources were determined by differences in their respective regression coefficients. The least significant difference method was used to test the differences in means among different treatments, and *p* < 0.05 was considered to be statistically significant.

## 3 Results

### 3.1 Growth performance and mortality

Compared with the control group, all Se-supplemented groups had no effect (*p* > 0.05) on the ADG, ADFI, FCR and mortality of broilers from 1 to 21 d of age (Table 1). The Se source, added Se level and their interaction had also no effects (*p* > 0.05) on the above indices.

**Table 1:** Effects of dietary Se source and level on growth performance and mortality of broilers from 1 to 21 d of age.

Item	Added Se level, mg/kg	ADG, g/d	ADFI, g/d	FCR, g/g	Mortality^4^, %
Control^1^	0.00	27.7	35.9	1.30	0.00
SS^1^	0.15	27.6	36.5	1.32	0.00
	0.30	26.2	34.9	1.33	0.00
	0.45	27.0	36.3	1.34	2.80
SY^1^	0.15	28.4	36.7	1.29	2.80
	0.30	28.1	36.1	1.29	2.80
	0.45	26.5	35.3	1.34	0.00
SM^1^	0.15	25.9	34.6	1.34	0.00
	0.30	27.7	36.2	1.31	0.00
	0.45	27.9	36.4	1.31	0.00
SO^1^	0.15	27.4	37.2	1.36	0.00
	0.30	26.4	34.6	1.31	0.00
	0.45	26.2	34.6	1.32	2.80
NS^1^	0.15	26.0	35.2	1.36	0.00
	0.30	28.6	37.6	1.32	0.00
	0.45	26.8	35.0	1.31	0.00
Pooled SEM		2.1	2.7	0.08	1.81
Se Source^2^	SS	27.0	35.9	1.33	0.93
	SY	27.7	36.0	1.31	1.85
	SM	27.1	35.7	1.32	0.00
	SO	26.7	35.5	1.33	0.93
	NS	27.1	35.9	1.33	0.00
Added Se level, mg/kg^3^	0.15	27.0	36.0	1.33	0.56
	0.30	27.4	35.9	1.31	0.56
	0.45	26.9	35.5	1.32	1.11
Pooled SEM		1.97	2.2	0.06	0.61
		*p*-value			
Se source		0.68	0.97	0.80	0.48
Added Se level		0.59	0.75	0.50	0.78
Source × level		0.12	0.27	0.88	0.54

^1^ Data represented the means of 6 replicates (*n* = 6).

^2^ Data represented the means of 18 replicates (*n* = 18).

^3^ Data represented the means of 30 replicates (*n* = 30).

^4 Percentage data for mortality of birds were transformed to arcsine for analysis.^

SS, sodium selenite; SY, selenium yeast; SM, selenomethionine; SO, hydroxyl-selenomethionine; NS, nano-selenium; ADG, average daily gain; ADFI, average daily feed intake; FCR, feed conversion ratio.

### 3.2 Se concentrations in plasma, tissues and erythrocyte

Compared with the control group, dietary supplemental Se increased (*p* < 0.05) Se concentrations in plasma, liver, breast muscle, pancreas, kidney and erythrocyte of broilers at 21 d of age (Table 2). The Se source, added Se level and their interactions affected (*p* < 0.05) Se concentrations in liver, breast muscle, pancreas, kidney and erythrocyte. The Se source and added Se level affected (*p* < 0.05) Se concentrations in plasma, but their interaction had no effect (*p* > 0.05) on the Se concentration in plasma. The plasma Se concentration in SS group was higher (*p* < 0.05) than that in SO and NS groups, but there were no differences (*p* > 0.05) among SS, SY and SM groups. Added 0.30 and 0.45 mg Se/kg had higher (*p* < 0.05) plasma Se concentration than added 0.15 Se/kg, while there was no difference (*p* > 0.05) between 0.30 and 0.45 Se/kg. No differences (*p* > 0.05) were observed in Se concentration in liver and breast muscle of broilers fed the SS diets among different added Se levels and in breast muscle of broilers fed the NS diets among different added Se levels; however, in SY, SM and SO groups, supplemental 0.45 mg Se/kg group had higher (*p* < 0.05) Se concentrations in liver and breast muscle than supplemental 0.30 and 0.15 mg Se/kg groups, and supplemental 0.30 mg Se/kg group had higher (*p* < 0.05) Se concentration than supplemental 0.15 mg Se/kg group. There was no difference (*p* > 0.05) in the Se concentration in pancreas of chicks fed different Se level of NS and between 0.15 and 0.30 mg Se/kg of SS groups, but the Se concentrations in pancreas of chicks fed the SY, SM and SO diets at 0.45 mg Se/kg were higher (*p* < 0.05) than those fed the diets at 0.30 and 0.15 mg Se/kg, and at 0.30 mg Se/kg was also higher (*p* < 0.05) than that at 0.15 mg/kg. No difference (*p* > 0.05) was found in the Se concentration in kidney of broilers fed the SY diets between 0.45 and 0.30 mg Se/kg, but supplemental 0.45 and 0.30 mg Se/kg groups were higher (*p* < 0.05) than those supplemental 0.15 mg Se/kg group. The Se concentration in kidney of broilers fed the SS, NS, SM and SO diets at 0.45 mg Se/kg was higher (*p* < 0.05) than those fed the diets at 0.30 and 0.15 mg Se/kg, and at 0.30 mg Se/kg was also higher (*p* < 0.05) than that at 0.15 mg Se/kg. There was no difference (*p* > 0.05) in Se concentration in erythrocyte between 0.45 and 0.30 mg Se/kg from NS group, but it was higher at 0.45 and 0.30 mg Se/kg than that at 0.15 mg Se/kg group (*p* < 0.05). The Se concentrations in erythrocyte from the SS, SY, SM and SO diets were higher (*p* < 0.05) in 0.45 mg Se/kg group than in 0.30 and 0.15 mg Se/kg groups, and in 0.30 mg Se/kg group than in 0.15 mg Se/kg group. As added Se level increased, the Se concentrations in plasma, liver, breast muscle, pancreas, kidney and erythrocyte increased linearly (*p* < 0.05).

**Table 2:** Effects of dietary Se source and level on Se concentrations in plasma, tissues and erythrocyte of broilers on d 21.

Item	Added Se level, mg/kg	Plasma, μg/mL	Liver^5^, μg/g	Breast muscle^5^, μg/g	Pancreas^5^, μg/g	Kidney ^5^, μg/g	Erythrocyte ^5^, μg/g
Control^1^	0.00	0.093^*^	0.135^*^	0.027^*^	0.109^*^	0.199^*^	0.055^*^
SS^1^	0.15	0.252	0.524^f^	0.074^fg^	0.207^i^	0.517^gh^	0.162^ij^
	0.30	0.259	0.538^ef^	0.078^fg^	0.218^hi^	0.562^def^	0.211^gh^
	0.45	0.281	0.548^def^	0.087^fg^	0.255^fg^	0.646^abc^	0.285^cd^
SY^1^	0.15	0.200	0.374^g^	0.096^fe^	0.203^i^	0.463^i^	0.154^j^
	0.30	0.257	0.582^cd^	0.143^c^	0.291^de^	0.648^ab^	0.241^ef^
	0.45	0.288	0.645^b^	0.199^b^	0.357^ab^	0.664^ab^	0.314^b^
SM^1^	0.15	0.187	0.399^g^	0.117^de^	0.232^gh^	0.516^gh^	0.185^hi^
	0.30	0.238	0.575^cde^	0.177^b^	0.317^dc^	0.593^de^	0.261^de^
	0.45	0.279	0.712^a^	0.241^a^	0.386^a^	0.688^ab^	0.355^a^
SO^1^	0.15	0.161	0.397^g^	0.099^fe^	0.212^i^	0.455^i^	0.149^j^
	0.30	0.239	0.600^c^	0.134^cd^	0.265^ef^	0.587^de^	0.218^fg^
	0.45	0.259	0.678^ab^	0.198^b^	0.324^bc^	0.691^a^	0.312^bc^
NS^1^	0.15	0.176	0.400^g^	0.066^g^	0.190^i^	0.485^hi^	0.119^k^
	0.30	0.219	0.512^f^	0.069^g^	0.204^i^	0.536^fg^	0.163^ij^
	0.45	0.229	0.515^f^	0.078^fg^	0.191^i^	0.601^cd^	0.185^hi^
Pooled SEM		0.012	0.006	0.002	0.003	0.006	0.003
Se source^2^	SS	0.264^a^	0.536	0.080	0.226	0.575	0.219
	SY	0.248^ab^	0.533	0.146	0.284	0.591	0.236
	SM	0.235^abc^	0.562	0.178	0.311	0.599	0.267
	SO	0.220^bc^	0.558	0.144	0.267	0.578	0.226
	NS	0.208^c^	0.476	0.071	0.195	0.541	0.156
Pooled SEM		0.009	0.006	0.002	0.004	0.014	0.008
Added Se level^3^, mg/kg	0.15	0.195^b^	0.419	0.090	0.209	0.487	0.154
	0.30	0.242^a^	0.551	0.120	0.259	0.585	0.219
	0.45	0.267^a^	0.619	0.161	0.303	0.658	0.290
Pooled SEM		0.010	0.009	0.005	0.005	0.008	0.005
	*P*-value						
Se source		0.03	<0.0001	<0.0001	<0.0001	0.0004	<0.0001
Added Se level		<0.0001	<0.0001	<0.0001	<0.0001	<0.0001	<0.0001
Source × level		0.80	<0.0001	<0.0001	<0.0001	<0.0001	0.0001
Linear effect^4^		<0.0001	<0.0001	<0.0001	<0.0001	<0.0001	<0.0001

^*^Different from all Se supplemental treatments by single degree of freedom contrast analysis (*P* < 0.05).

^a-j^Means with different superscripts within the same column differ (*P* < 0.05).

^1^Data represented the means of 6 replicates (*n* = 6).

^2^Data represented the means of 18 replicates (*n* = 18).

^3^Data represented the means of 30 replicates (*n* = 30).

^4Linear effects of added Se level.^

^5Fresh tissue basis.^

SS, sodium selenite; SY, selenium yeast; SM, selenomethionine; SO, hydroxyl-selenomethionine; NS, nano-selenium.

### 3.3 GPX and DIO activities in plasma and tissues

Compared with the control group, dietary supplemental Se increased (*p* < 0.05) the GPX activities in plasma, liver, breast muscles, pancreas and kidney of chicks at 21 d of age (Table 3). The Se source affected (*p* < 0.05) GPX activities in breast muscle, pancreas and kidney, but it did not affect (*p* > 0.05) the GPX activities in plasma and liver. Added Se level affected (*p* < 0.05) the GPX activities in plasma, liver, breast muscles, pancreas and kidney, but the interaction between Se source and added Se level did not affect (*p* > 0.05) them. The broilers fed the diets supplemented with 0.30 and 0.45 mg Se/kg had higher (*p* < 0.05) GPX activities in plasma, liver and kidney than those fed the diet supplemented with 0.15 mg Se/kg, whereas there was no difference (*p* > 0.05) between supplemental 0.30 and 0.45 mg Se/kg groups. The GPX activities in pancreas and breast muscle of broilers fed the diet at 0.45 mg Se/kg were higher (*p* < 0.05) than those fed the diets at 0.30 and 0.15 Se/kg, and at 0.30 mg Se/kg were higher (*p* < 0.05) than those at 0.15 mg Se/kg. The broilers fed the SM diet had the greatest (*p* < 0.05) GPX activity in pancreas than those fed the SS, NS and SO diets, but there was no difference (*p* > 0.05) between SY and SM. However, the broilers fed the SS diet had the greatest (*p* < 0.05) GPX activity in kidney than those fed the SY, SM and SO diets, but no difference (*p* > 0.05) was observed between SS and NS; furthermore, the chicks fed NS diet had higher (*p* < 0.05) GPX activity in kidney than those fed the SO diet. The chicks fed the SS, SY and SM diets had higher (*p* < 0.05) GPX activity in breast muscle than those fed the SO diet, while there were no differences (*p* > 0.05) among SM, SY and SS groups. As added Se level increased, the GPX activities in plasma, liver, pancreas, breast muscle and kidney increased linearly (*p* < 0.05).

**Table 3:** Effects of dietary Se source and level on glutathione peroxidase (GPX) activity in plasma and tissues of broilers on d 21.

Item	Added Se level, mg/kg	Plasma, U/mL	Liver, U/mg protein	Pancreas, U/mg protein	Kidney, U/mg protein	Breast muscle, U/mg protein
Control^1^	0.00	155*	6.31*	2.00*	3.68*	0.00*
SS^1^	0.15	1,433	26.0	2.50	19.9	6.28
	0.30	1,484	26.6	4.07	22.1	9.50
	0.45	1,559	27.3	4.88	22.9	9.92
SY^1^	0.15	1,166	17.9	3.13	14.7	3.33
	0.30	1,473	24.3	5.99	20.1	8.97
	0.45	1,532	25.4	8.83	21.7	11.97
SM^1^	0.15	1,036	17.1	3.99	14.9	4.42
	0.30	1,440	26.7	6.91	19.2	10.6
	0.45	1,495	28.5	9.79	20.7	10.8
SO^1^	0.15	1,087	18.7	2.30	12.9	1.51
	0.30	1,458	25.2	4.19	18.5	6.47
	0.45	1,471	29.9	8.40	19.4	10.1
NS^1^	0.15	1,326	22.2	2.75	16.1	4.45
	0.30	1,479	26.4	4.08	20.9	7.50
	0.45	1,518	29.7	4.74	22.7	8.46
Pooled SEM		80.3	0.784	0.325	0.545	0.41
Se source^2^	SS	1,492	26.6	3.82^c^	21.6^a^	8.57^a^
	SY	1,390	22.5	5.98^ab^	18.8^bc^	8.09^ab^
	SM	1,324	24.1	6.90^a^	18.3^bc^	8.59^a^
	SO	1,339	24.6	4.96^bc^	16.9^c^	6.04^c^
	NS	1,441	26.1	3.86^c^	19.9^ab^	6.81^bc^
Pooled SEM		48.3	1.13	0.43	0.86	0.60
Added Se level^3^, mg/kg	0.15	1210^b^	20.4^b^	2.93^c^	15.7^b^	4.00^c^
	0.30	1467^a^	25.8^a^	5.05^b^	20.1^a^	8.60^b^
	0.45	1515^a^	28.2^a^	7.33^a^	21.5^a^	10.3^a^
Pooled SEM		41.3	0.876	0.292	0.53	0.45
	*P*-value					
Se source		0.21	0.09	0.0014	0.0001	0.008
Added Se level		<0.0001	<0.0001	<0.0001	<0.0001	<0.0001
Source × Level		0.67	0.25	0.41	0.83	0.13
Linear effect^4^		<0.0001	<0.0001	<0.0001	<0.0001	<0.0001

^*^Different from all Se supplemental treatments by single degree of freedom contrast analysis (*P* < 0.05).

^a-c^Means with different superscripts within the same column differ (*P* < 0.05).

^1^Data represented the means of 6 replicates (*n* = 6).

^2^Data represented the means of 18 replicates (*n* = 18).

^3^Data represented the means of 30 replicates (*n* = 30).

^4Linear effects of added Se level.^

SS, sodium selenite; SY, selenium yeast; SM, selenomethionine; SO, hydroxyl-selenomethionine; NS, nano-selenium; GPX, glutathione peroxidase.

Compared with the control group, all Se-supplemented groups had no effect (*p* > 0.05) on the DIO activities in plasma, liver and pancreas of broilers on d 21 (Table 4). The Se source, Se level and their interactions had also no effects (*p* > 0.05) on these indices.

**Table 4:** Effects of dietary Se source and level on iodothyronine deiodinase (DIO) activity in plasma and tissues of broilers on d 21.

Item	Added Se level, mg/kg	Plasma, U/L	Liver, U/g protein	Pancreas, U/g protein
Control^1^	0.00	2.133	0.226	0.114
SS^1^	0.15	2.028	0.196	0.097
	0.30	1.690	0.190	0.088
	0.45	1.775	0.189	0.090
SY^1^	0.15	2.226	0.194	0.083
	0.30	2.278	0.192	0.124
	0.45	2.110	0.195	0.122
SM^1^	0.15	2.108	0.196	0.087
	0.30	2.245	0.219	0.097
	0.45	2.365	0.210	0.095
SO^1^	0.15	1.947	0.188	0.088
	0.30	1.722	0.189	0.087
	0.45	1.865	0.226	0.105
NS^1^	0.15	2.085	0.224	0.112
	0.30	2.055	0.224	0.083
	0.45	2.118	0.218	0.100
Pooled SEM		0.100	0.005	0.015
Se source^2^	SS	1.843	0.192	0.092
	SY	2.211	0.194	0.108
	SM	2.239	0.208	0.093
	SO	1.843	0.201	0.093
	NS	2.084	0.222	0.098
Pooled SEM		0.087	0.007	0.010
Added Se level^3^, mg/kg	0.15	2.073	0.200	0.093
	0.30	1.999	0.203	0.095
	0.45	2.057	0.207	0.102
Pooled SEM		0.099	0.006	0.009
	*P* -value			
Se source		0.10	0.11	0.88
Added Se level		0.86	0.73	0.81
Source × Level		0.97	0.76	0.94

^1^Data represented the means of 6 replicates (*n* = 6).

^2^Data represented the means of 18 replicates (*n* = 18).

^3^Data represented the means of 30 replicates (*n* = 30).

SS, sodium selenite; SY, selenium yeast; SM, selenomethionine; SO, hydroxyl-selenomethionine; NS, nano-selenium; DIO, iodothyronine deiodinase.

### 3.4 mRNA expressions of selenproteins in tissues

Compared with the control chicks, the chicks fed Se-supplemented diets had higher (*p* < 0.05) *Gpx1*, *Gpx4*, *Selenou*, *Selenop* and *Dio1* mRNA expressions in the liver, and *Gpx1*, *Gpx4* and *Selenou* mRNA expressions in the pancreas. However, there were no differences (*p* > 0.05) in these selenprotein mRNA expressions in breast muscles and kidney, and the *Selenop* and *Dio1* mRNA expressions in the pancreas (Tables 5, 6). The Se source affected (*p* < 0.05) the *Selenop* mRNA expression in liver, but added Se level and the interaction between Se source and added Se level had no effects (*p* > 0.05) on *Selenop* mRNA expression. Additionally, the Se source, added Se level and their interaction had no effects (*p* > 0.05) on the *Gpx1*, *Gpx4*, *Selenou* and *Dio1* mRNA expressions in liver. The Se source, added Se level and their interaction did not affect (*p* > 0.05) all the selenprotein mRNA expressions in the breast muscles and kidney. However, added Se level affected (*p* < 0.05) the *Gpx1*, *Selenop* and *Dio1* mRNA expressions in pancreas, but did not affect (*p* > 0.05) *Gpx4* and *Selenou* mRNA expressions. The Se source and the interaction between Se source and added Se level had no effect (*p* > 0.05) on these mRNA expressions in pancreas. Specifically, the *Selenop* mRNA expression in liver of chicks fed the SS diet was higher (*p* < 0.05) than those fed the SO diet, but there were no differences (*p* > 0.05) between SS and all other treatments. The chicks fed the diets supplemented with 0.45 and 0.30 mg Se/kg had higher (*p* < 0.05) *Gpx1* and *Dio1* mRNA expressions in pancreas than those fed the diet supplemented with 0.15 mg Se/kg, but there was no difference (*p* > 0.05) between 0.45 and 0.30 mg Se/kg groups. The *Selenop* mRNA expression in 0.45 Se/kg group was higher (*p* < 0.05) than that in 0.15 Se/kg group, but no differences (*p* > 0.05) were observed between 0.30 Se/kg group and 0.45 or 0.15 Se/kg groups. As added Se level increased, the mRNA expressions of *Gpx1* and *Selenop* in pancreas increased linearly (*p* < 0.05).

**Table 5:** Effects of dietary Se source and level on mRNA expression level (RQ) of selenproteins in liver and breast muscle of broilers on d 21.

Item	Added Se level, mg/kg	Liver					Breast muscle				
		Gpx1	Gpx4	Selenou	Selenop	Diol	Gpx1	Gpx4	Selenou	Selenop	Diol
Control^1^	0.00	1.00*	1.00*	1.00*	1.00*	1.00*	1.00	1.00	1.00	1.00	1.00
SS^1^	0.15	2.20	2.61	2.62	2.45	1.64	1.42	1.29	1.21	1.36	0.84
	0.30	2.57	2.43	3.16	2.64	1.97	1.28	1.21	1.12	1.41	1.30
	0.45	2.48	2.41	2.95	2.30	2.14	1.34	1.05	1.08	1.29	0.86
SY^1^	0.15	1.62	1.97	2.46	2.03	1.45	1.21	1.33	1.28	1.30	0.96
	0.30	2.21	2.38	2.93	2.28	1.93	1.49	1.26	1.14	1.37	0.99
	0.45	2.11	2.11	2.82	2.31	1.67	1.14	1.03	1.12	1.17	0.74
SM^1^	0.15	2.08	2.21	2.55	2.07	1.98	1.10	1.09	1.13	1.19	0.64
	0.30	2.11	2.35	2.39	2.29	1.65	1.40	1.21	1.27	1.32	1.01
	0.45	2.04	2.27	2.45	2.17	1.76	1.48	1.09	1.57	1.38	1.05
SO^1^	0.15	1.30	1.88	2.03	1.84	1.47	1.29	1.19	1.33	1.37	0.63
	0.30	1.98	1.99	2.26	1.82	1.57	1.24	1.11	1.25	1.17	1.20
	0.45	2.62	2.59	2.98	2.03	2.11	1.27	0.98	1.37	1.08	0.96
NS^1^	0.15	1.86	2.02	2.88	1.98	1.87	1.18	1.21	1.11	1.23	1.14
	0.30	2.17	2.53	3.16	2.25	2.06	1.40	1.18	1.15	1.14	0.78
	0.45	1.80	2.21	2.95	2.37	1.77	1.03	1.26	1.21	1.24	1.36
Pooled SEM		0.22	0.09	0.16	0.10	0.08	0.08	0.05	0.13	0.06	0.08
Se source^2^	SS	2.41	2.48	2.91	2.47^a^	1.91	1.35	1.18	1.13	1.35	1.01
	SY	1.97	2.15	2.74	2.21^ab^	1.69	1.28	1.20	1.18	1.28	0.89
	SM	2.08	2.27	2.46	2.18^ab^	1.80	1.33	1.30	1.33	1.30	0.90
	SO	2.01	2.17	2.45	1.9^b^	1.73	1.27	1.09	1.32	1.21	0.95
	NS	1.94	2.25	2.99	2.2^ab^	1.90	1.20	1.22	1.16	1.21	1.11
Pooled SEM		0.23	0.10	0.15	0.08	0.09	0.07	0.07	0.09	0.08	0.11
Added Se level^3^, mg/kg	0.15	1.83	2.15	2.52	2.08	1.69	1.24	1.22	1.21	1.29	0.85
	0.30	2.21	2.33	2.78	2.26	1.83	1.36	1.19	1.19	1.29	1.07
	0.45	2.21	2.32	2.83	2.24	1.89	1.25	1.08	1.28	1.23	0.99
Pooled SEM		0.18	0.07	0.10	0.08	0.07	0.07	0.05	0.08	0.08	0.09
Se source		0.70	0.25	0.052	0.02	0.51	0.83	0.80	0.66	0.89	0.78
Added Se level		0.30	0.24	0.16	0.26	0.23	0.42	0.26	0.78	0.88	0.29
Source × Level		0.92	0.24	0.60	0.87	0.17	0.38	0.54	0.94	0.93	0.98

^*^Different from all Se supplemental treatments by single degree of freedom contrast analysis (*P* < 0.05).

^a,b^Means with different superscripts within the same column differ (*P* < 0.05).

^1^The mRNA expression levels were calculated as the relative quantity (RQ) of the target gene mRNA to the geometric mean of β-actin mRNA and GAPDH, RQ = 2^–ΔΔCT^ (CT = threshold cycle).

^2^Data represented the means of 6 replicates (*n* = 6).

^3^Data represented the means of 18 replicates (*n* = 18).

^4^Data represented the means of 30 replicates (*n* = 30).

SS, sodium selenite; SY, selenium yeast; SM, selenomethionine; SO, hydroxyl-selenomethionine; NS, nano-selenium; Gpx1, glutathione peroxidase 1; Gpx4, glutathione peroxidase 4; Selenop, selenoprotein P; Selenou, Selenoprotein U; Dio1, iodothyronine deiodinase 1.

**Table 6:** Effects of dietary Se source and level on mRNA expression level (RQ) of selenproteins in pancreas and kidney of broilers on d 21.

Item	Added Se level, mg/kg	Pancreas					Kidney				
		*Gpx1*	*Gpx4*	*Selenou*	*Selenop*	*Diol*	*Gpx1*	*Gpx4*	*Selenou*	*Selenop*	*Diol*
Control^1^	0.00	1.00*	1.00*	1.00*	1.00	1.00	1.00	1.00	1.00	1.00	1.00
SS^1^	0.15	1.86	2.35	2.88	1.44	0.52	2.55	1.56	1.61	2.41	1.57
	0.30	1.56	2.53	2.71	1.41	1.78	1.10	0.95	1.79	2.04	1.43
	0.45	1.36	1.54	2.24	1.26	0.77	1.54	1.33	1.62	2.19	1.29
SY^1^	0.15	1.15	1.72	2.11	1.17	0.68	1.21	1.40	1.61	2.07	1.29
	0.30	1.80	2.01	2.66	1.76	1.15	1.67	1.65	2.52	1.92	1.22
	0.45	1.98	2.47	2.65	2.40	1.70	1.81	1.46	1.88	1.93	1.24
SM^1^	0.15	1.37	2.12	2.72	1.55	0.52	0.67	1.26	1.48	1.84	1.15
	0.30	1.51	2.01	2.56	1.64	0.94	1.34	1.31	1.59	1.99	1.17
	0.45	1.93	1.98	2.99	1.97	1.25	0.79	1.48	1.59	2.13	1.10
SO^1^	0.15	1.50	1.91	2.74	1.29	0.97	1.29	1.63	1.51	1.44	1.16
	0.30	2.35	2.10	2.59	1.96	1.43	1.52	1.21	1.56	1.77	1.15
	0.45	2.11	2.11	3.09	2.48	0.82	1.64	1.39	1.74	1.96	1.36
NS^1^	0.15	1.17	1.61	1.63	1.35	0.98	1.24	1.19	1.49	1.88	1.16
	0.30	1.51	2.07	2.63	1.88	1.32	1.38	1.46	1.58	1.95	1.32
	0.45	1.66	2.31	2.34	2.66	1.31	0.66	0.77	0.92	1.03	0.72
Pooled SEM		0.10	0.10	0.11	0.10	0.05	0.05	0.03	0.07	0.08	0.03
Se source^2^	SS	1.59	2.14	2.61	1.37	1.05	1.72	1.30	1.67	2.20	1.42
	SY	1.64	2.07	2.47	1.78	1.18	1.55	1.50	2.00	1.98	1.25
	SM	1.61	2.03	2.76	1.72	0.91	0.96	1.35	1.56	1.99	1.14
	SO	1.99	2.04	2.81	1.94	1.09	1.50	1.40	1.61	1.76	1.23
	NS	1.45	2.00	2.20	1.96	1.21	1.12	1.16	1.35	1.66	1.09
Pooled SEM		0.09	0.13	0.16	0.15	0.14	0.17	0.19	0.23	0.28	0.17
Added Se level^3^, mg/kg	0.15	1.41^b^	1.94	2.42	1.36^b^	0.74^c^	1.38	1.40	1.54	1.95	1.26
	0.30	1.75^a^	2.14	2.63	1.73^ab^	1.33^a^	1.41	1.33	1.81	1.94	1.26
	0.45	1.81^a^	2.08	2.66	2.15^a^	1.18^ab^	1.31	1.30	1.57	1.88	1.16
Pooled SEM		0.10	0.11	0.14	0.10	0.08	0.18	0.16	0.19	0.24	0.15
Se source		0.14	0.99	0.33	0.52	0.51	0.18	0.82	0.59	0.79	0.81
Added Se level		0.04	0.60	0.56	0.02	0.02	0.91	0.87	0.63	0.97	0.84
Source × Level		0.23	0.29	0.60	0.82	0.34	0.54	0.38	0.89	0.98	0.98
Linear effect^4^		0.002			0.02	0.38					

^*^Different from all Se supplemental treatments by single degree of freedom contrast analysis (*P* < 0.05).

^a-c^Means with different superscripts within the same column differ (*P* < 0.05).

^1^The mRNA expressions were calculated as the relative quantity (RQ) of the target gene mRNA to the geometric mean of β-actin mRNA and GAPDH, RQ = 2^–ΔΔCT^ (CT = threshold cycle).

^1^Data represented the means of 6 replicates (*n* = 6).

^2^Data represented the means of 18 replicates (*n* = 18).

^3^Data represented the means of 30 replicates (*n* = 30).

^4Linear effects of added Se level.^

SS, sodium selenite; SY, selenium yeast; SM, selenomethionine; SO, hydroxyl-selenomethionine; NS, nano-selenium; Gpx1, glutathione peroxidase 1; Gpx4, glutathione peroxidase 4; Selenop, selenoprotein P; Selenou, Selenoprotein U; Dio1, iodothyronine deiodinase 1.

### 3.5 Estimation of the relative bioavailabilities of se from SY, SM, SO, and NS

Regressions were calculated based on daily dietary Se intake during the experimental period (Table 7). Multiple linear regression relationships (*p* < 0.05) were observed in the Se concentrations in plasma, liver, breast muscle, kidney and erythrocyte, GPX activities in plasma and these tissues, and *Gpx*1 and *Selenop* mRNA expressions in pancreas of broiler chicks. However, owing to the R^2^ values were relatively lower in *Gpx*1 (*R*^2^ = 0.30) and *Selenop* (*R*^2^ = 0.15) mRNA expressions than other indices, the relative bioavailability values (Table 8) were estimated according to the above other indices except for these two indices. No differences (*p* > 0.05) in slopes between SS and SY or SM or SO or NS were observed in Se concentrations in plasma and kidney, GPX activities in plasma, liver and breast muscle. However, the differences (*p* < 0.05) in slopes between SS and SY or SM or SO or NS were observed in the Se concentrations in liver, breast muscle, pancreas and erythrocyte, GPX activities in pancreas and kidney. Based on slope ratios from the multiple linear regressions of the above indices, the Se bioavailabilities of SY, SM, SO, NS relative to SS (100%) were 78 to 367%, 67.8 to 471%, 57 to 372%, and 45 to 92%, respectively.

**Table 7:** Multiple linear regressions of Se concentrations in plasma, tissues and erythrocyte, glutathione peroxidase (GPX) activities in plasma and tissues, and *Gpx1* and selenoprotein P (*Selenop*) mRNA expressions in pancreas based on daily dietary Se intake^1^.

Dependent variables	Regression equation^2^	*R* ^2^	*P*-value
Plasma Se	Y = 0.1651 + 0.01049×_1_ + 0.01008×_2_ + 0.008282×_3_ + 0.007343×_4_ + 0.004725×_5_	0.51	<0.0001
Liver Se	Y = 0.3232 + 0.02139×_1_ + 0.02597×_2_ + 0.02879×_3_ + 0.02986×_4_ + 0.01661×_5_	0.94	<0.0001
Breast muscle Se	Y = 0.05113 + 0.003104×_1_ + 0.01140×_2_ + 0.01462×_3_ + 0.01154×_4_ + 0.002076×_5_	0.91	<0.0001
Pancreas Se	Y = 0.1597 + 0.007631×_1_ + 0.01525×_2_ + 0.01752×_3_ + 0.01332×_4_ + 0.003446×_5_	0.87	<0.0001
Kidney Se	Y = 0.4025 + 0.01966×_1_ + 0.02274×_2_ + 0.02230×_3_ + 0.02250×_4_ + 0.01511×_5_	0.91	<0.0001
Erythrocyte Se	Y = 0.08134 + 0.01595×_1_ + 0.01856×_2_ + 0.02129×_3_ + 0.01812×_4_ + 0.00823×_5_	0.91	<0.0001
Plasma GPX activity	Y = 1,081 + 44.2×_1_ + 38.2×_2_ + 31.9×_3_ + 33.3×_4_ + 37.5×_5_	0.71	<0.0001
Liver GPX activity	Y = 16.8 + 1.02×_1_ + 0.71×_2_ + 0.91×_3_ + 1.02×_4_ + 1.03×_5_	0.65	<0.0001
Breast muscle GPX activity	Y = 1.1334 + 0.8125×_1_ + 0.8613×_2_ + 0.8476×_3_ + 0.6605×_4_ + 0.6164×_5_	0.70	<0.0001
Pancreas GPX activity	Y = 0.6636 + 0.3592×_1_ + 0.6480×_2_ + 0.7174×_3_ + 0.5504×_4_ + 0.3319×_5_	0.51	<0.0001
Kidney GPX activity	Y = 13.1 + 0.91×_1_ + 0.71×_2_ + 0.62×_3_ + 0.52×_4_ + 0.77×_5_	0.78	<0.0001
Pancreas *Gpx1* mRNA	Y = 1.3220 + 0.01712×_1_ + 0.04477×_2_ + 0.03625×_3_ + 0.08109×_4_ + 0.01881×_5_	0.30	0.009
Pancreas *Selenop* mRNA	Y = 1.01592 + 0.03221×_1_ + 0.09633×_2_ + 0.07449×_3_ + 0.11493×_4_ + 0.11099×_5_	0.15	0.026

^1Regression analyses were based on cage averages with 6 cages per Se source.^

^2Y represents Se concentrations in plasma, liver, breast muscle, pancreas, kidney, and erythrocyte, GPX activities in plasma, liver, breast muscle, pancreas, and kidney, and pancreas Gpx1 and Selenop mRNA expressions, respectively; X1, X2, X3, X4, and X5 represents daily dietary analyzed Se intake of SS, SY, SM, SO, and NS, respectively.^

SS, sodium selenite; SY, selenium yeast; SM, selenomethionine; SO, hydroxyl-selenomethionine; NS, nano-selenium.

**Table 8:** Relative bioavailability values (RBV) of different Se sources based on slope ratios from multiple linear regressions of Se concentrations in plasma, tissues and erythrocyte, glutathione peroxidase (GPX) activities in plasma and tissues on daily dietary analyzed Se intake^1^.

Dependent variables	Regression coefficients, slope (SE)					RBV, 100% (SE)				
	SS	SY	SM	SO	NS	SS	SY	SM	SO	NS
Plasma Se	0.010(0.002)	0.010(0.002)	0.008(0.002)	0.007(0.002)	0.005(0.002)	100	96(18)	80 (17)	70 (18)	45 (16)
Liver Se	0.021(0.002)^bc^	0.026(0.002)^ab^	0.029(0.002)^a^	0.030(0.002)^a^	0.017(0.002)^cd^	100	121(11)	135(12)	140(13)	78(9)
Breast muscle Se	0.003(0.001)^c^	0.011(0.001)^b^	0.015(0.001)^a^	0.012(0.001)^b^	0.002(0.001)^c^	100	367(82)	471(109)	372(83)	67(20)
Pancreas Se	0.008(0.001)^c^	0.015(0.001)^ab^	0.018(0.001)^a^	0.013(0.001)^b^	0.003(0.001)^d^	100	200(26)	230(30)	175(22)	45 (12)
Kidney Se	0.020(0.002)	0.023(0.002)	0.022(0.002)	0.023(0.002)	0.015(0.002)	100	116(10)	113(9)	114(10)	77(8)
Erythrocyte Se	0.016(0.001)^b^	0.019(0.001)^ab^	0.021(0.001)^a^	0.018(0.001)^b^	0.008(0.001)^c^	100	116(7)	133(8)	114(7)	52(5)
Plasma GPX	44.2(9.63)	38.2(9.88)	31.9(9.35)	33.3(10.10)	37.5(9.14)	100	86(19)	72.3(18)	75 (19)	85(18)
Liver GPX	1.02(0.195)	0.71(0.200)	0.91(0.189)	1.02(0.204)	1.03(0.185)	100	70(16)	89.4(17)	100(18)	101(17)
Breast muscle GPX	0.813(0.106)	0.861(0.109)	0.848(0.103)	0.661(0.111)	0.616(0.100)	100	106(13)	104(12)	81 (12)	76(11)
Pancreas GPX	0.359(0.097)^b^	0.648(0.100)^a^	0.717(0.094)^a^	0.550(0.102)^ab^	0.332(0.092)^b^	100	180(40)	200(45)	153(34)	92 (23)
Kidney GPX	0.909(0.119)^a^	0.710(0.122)^ab^	0.616(0.115)^ab^	0.520(0.124)^bc^	0.766(0.113)^ab^	100	78(11)	67.8(11)	57 (11)	84 (11)

^a–d^ Means with different superscripts within the same row differ (*P* < 0.05).

^1Regression analyses of Se concentrations in plasma, liver, breast muscle, pancreas, kidney, and erythrocyte, GPX activities in plasma, liver, breast muscle, pancreas and kidney of broilers on d 21 were based on cage averages with 6 cages per Se source and daily dietary analyzed Se intake.^

SS, sodium selenite; SY, selenium yeast; SM, selenomethionine; SO, hydroxyl-selenomethionine; NS, nano-selenium.

## 4 Discussion

Our study has demonstrated that the Se concentrations in liver, breast muscle, pancreas and erythrocyte, and GPX activities in kidney and pancreas of broilers could be used as sensitive indices to evaluate the bioavailability of different Se sources. The new parameters were obtained for the relative bioavailabilities of Se from SY, SM, SO and NS. The Se from SM, SY and SO are more available to broilers than the Se from SS, and the Se from SM had the highest while the Se from NS had the lowest relative bioavailability. It is of great significance to find sensitive indices to evaluate the Se nutritional status and bioavailability for different Se sources in the growth and metabolic regulation of broiler chickens. These findings provide a scientific basis for the application of different Se source additives in the broiler production.

Many studies have shown that the Se from SY and SM could increase the Se concentrations in muscle of animals compared with the Se from SS (33–37). Organic forms of Se, such as SM, could directly replace methionine to participate in protein synthesis, so it is easier to deposit in muscle. However, selenite can only be reduced to hydrogen selenide and then converted to selenoproteins, which depending on the activity of TXNRD (38–40). Although SS and NS can also promote the synthesis of selenoproteins, they lack the metabolic pathway to transform into SM, and can hardly improve the Se concentrations in muscle (41–43). Therefore, the Se concentration in breast muscle of chickens in this study was higher in organic SM, SO and SY treatments than those in SS and NS treatments, which may be associated with the mode of intestinal absorption and the ability to be incorporated into proteins in the place of methionine. The regularity of the Se concentrations in pancreas was in agreement with that in breast muscle, which was also the highest in SM treatment, suggesting that Se in the form of SM was easier to deposit in tissues. In addition, the liver is the metabolic center organ for Se regulation and also the main synthesis site of selenoenzymes and selenoproteins, which could regulate whole-body Se by producing excretory metabolites and distributes Se to other tissues by secreting Selenop into the plasma (2, 8). Compared with other treatments, the Se content in liver was lower in NS treatment. These discrepancy deposition among different Se sources might be explained by the absorption process and metabolic pathways (44). It has been found that selenoamino acids are effectively transported by various intestinal amino acid transporters and are thus available for Se metabolism (45, 46). Additionally, many studies have demonstrated that the deposition efficiency of NS is lower than that of SY or SM (44, 47). However, the exact mechanisms for NS absorption and metabolic pathways need to be further investigated. The Se concentration in liver from SS and NS treatments was higher than that from organic Se treatments at 0.15 mg/kg low Se level. However, with the increase of dietary Se level, it is difficult for inorganic Se to reach the liver Se content as organic Se treatments, which may be related to their different metabolic regulation in the body (48). The absorbed Se was transported into the plasma to various organs to be utilized and the redundant Se was also transported in the plasma into excretion organs. In the SS treatment group, the Se concentration in this study was higher in plasma but lower in the breast muscle, indicating that the Se in the form of SS has a lower bioavailability in improving the Se content in tissues. In kidney, the synthesis of selenoproteins are active and the redundant Se that could not be stored in the body and thus was excreted through the kidney. The present study demonstrated that the Se content in kidney is higher than that in other organs. It is worth mentioning that the Se concentration in erythrocyte was also determined in the current study. The Se deficiency can cause the decrease of antioxidant ability in erythrocyte, and a small amount of SS can help erythrocyte keep alive when human erythrocyte is preserved outside of the body. The results from the present study showed that the Se concentration of erythrocyte in SS and NS treatments were lower than that in SM, SY, and SO treatments, which is consistent with the regularity in the tissues, indicating that organic Se is also more easy to be deposited in erythrocyte.

In the present study, all Se-supplemented groups could increase the activity of GPX in plasma and tissues compared with the control group, indicating that different Se sources could participate in the synthesis of GPX. Nevertheless, the results from this study demonstrated that different Se sources had different effects on GPX activities in breast muscle, pancreas and kidney of broilers. The chicks fed the Se from SM had the highest GPX activities in pancreas and breast muscle, and the Se from SS, SO and NS in pancreas as well as SO and NS in breast muscle had relatively lower GPX activities. However, in the other organ, a part of inorganic Se which could not be deposited into proteins in a non-specific way was excreted through kidney, which might be the reason that the GPX activity in kidney in SS and NS treatments were higher than that in SM, SY and SO treatments. The GPX3 is the only secretory GPX in the body, so it is also called plasma GPX. The GPX3 is mainly derived from the kidney and is produced and released into the blood by epithelial cells of proximal renal tubules and Bowman’s capsule wall (49). The results in the present study showed that the regularity of GPX activity in plasma of different treatments was consistent with that in kidney. Additionally, the mRNA expressions of *Gpx*1 and *Selenop* in pancreas increased linearly with the increase of Se level, but the R^2^ values in the regression analysis results were relatively low for *Gpx*1 and *Selenop*, so they were not suitable for analysis of the relative Se bioavailability. However, our previous study showed that both GPX mRNA expression and activity in pancreas of chicks fed 0.20 and 0.40 mg Se/kg were sensitive indices to evaluate the bioavailability of SY (16). These inconsistent results for mRNA expression might be due to different supplemental Se levels (0.15, 0.30 and 0.45 mg Se/kg) in the experiment diets for the present study, which might influence the sensitivity of selenoprotein expressions.

The results from this present study showed that the bioavailabilities of organic Se from SM, SY and SO were higher than that from SS due to their higher absorption and deposition rate, which is consistent with many previous studies (10, 12, 13, 17, 33). However, when the kidney GPX activity was used as the evaluation index, the bioavailability of SO treatment was lower than that of SS treatment, which may be related to the metabolic difference of SO in the body. As we have discussed before, the redundant Se that could not be stored in the body was excreted through the kidney, so the lower GPX activity in the kidney probably means the less excreted Se level in the kidney. Recent study have shown that NS has higher biological activity, safety and antioxidant capacity than SS (14). However, the results of this study showed that the bioavailability of NS was significantly lower than that of SS using the Se concentration in erthrocyte and pancreas as evaluation indices, which may be related to the product quality from different companies and the absorption of NS in the intestine. The absorption pattern of NS has not been reported yet, and whether its absorption rate is lower than that of SS remains further study.

## 5 Conclusion

The results from this study indicated that the Se from SM, SY, and SO are more available to broilers than the Se from SS in enhancing the Se concentrations in liver, breast muscle, pancreas and erythrocytes and GPX activity in pancreas, and the Se from SM had the highest while the Se from NS had the lowest relative bioavailability.

## Data Availability

The original contributions presented in the study are included in the article/supplementary material, further inquiries can be directed to the corresponding author/s.
