# Supplementary material for: Relative bioavailability of selenium yeast, selenomethionine, hydroxyl-selenomethionine and nano-selenium for broilers
Source: Front Vet Sci. 2025 Jan 17;11:1542557. doi: 10.3389/fvets.2024.1542557 (PMC11782124; doi:10.3389/fvets.2024.1542557)
Supplement: Supplementary file 1 [file Table_1.DOC]

**Supplementary TABLE S1** Composition and nutrient levels of the basal diet for broilers from 1 to 21 d of age (as-fed basis)

| **Item** | **Content** |
| --- | --- |
| Ingredients, % |  |
| Corn | 53.46 |
| Soybean meal | 37.45 |
| Soybean oil | 5.00 |
| CaHPO_4_^1^ | 1.70 |
| CaCO_3_^1^ | 1.28 |
| Sodium chloride^1^ | 0.30 |
| DL-Met^1^ | 0.27 |
| Micronutrients^1, 2^ | 0.34 |
| Corn starch +Se additive^3^ | 0.20 |
| Nutrient levels |  |
| ME, kcal/kg | 3000 |
| Crude protein^4^,% | 21.69 |
| Lysine, % | 1.14 |
| Methionine, % | 0.61 |
| Methionine +Cysteine,% | 0.90 |
| Calcium^4^,% | 1.00 |
| Non-phytate phosphorus,% | 0.45 |
| Se^4^, mg/kg | 0.02 |

^1^*Reagent grade*.

^2^ *Supplied per kilogram of diet: 15,000 IU vitamin A (all trans-retinol acetate); 4,500 IU cholecalciferol; 24 IU vitamin E (all-rac-α-tocopherol acetate); 3 mg vitamin K (menadione sodium bisulfate); 3 mg thiamin (thiamin mononitrate); 9.6 mg riboflavin; 3 mg vitamin B_6_; 0.018 mg vitamin B_12_; 15 mg calcium pantothenate; 39 mg niacin; 1.5 mg folic acid; 0.15 mg biotin; 700 mg choline (choline chloride); 8 mg Cu (CuSO_4_ 5H_2_O); 40 mg Fe (FeSO_4_·7H_2_O); 110 mg Mn (MnSO_4_·H_2_O); 60 mg Zn (ZnSO_4_·7H_2_O); 0.35 mg I (KI)*.

^3^ *Se additives, different Se sources were added in place of the equivalent weight of corn starch*.

^4^ *Analyzed values based on triplicate determinations, and the others were calculated values*.

**Supplementary TABLE S2** Analyzed Se concentrations in different treatment diets for broilers

| **Se source** | **Added Se level, mg/kg** | **Analyzed Se concentrations in diets^1^, mg/kg (air-dry basis)** |
| --- | --- | --- |
| Control | 0.00 | 0.020 |
| SS | 0.15 | 0.169 |
|  | 0.30 | 0.309 |
|  | 0.45 | 0.468 |
| SY | 0.15 | 0.177 |
|  | 0.30 | 0.301 |
|  | 0.45 | 0.453 |
| SM | 0.15 | 0.161 |
|  | 0.30 | 0.317 |
|  | 0.45 | 0.463 |
| SO | 0.15 | 0.162 |
|  | 0.30 | 0.312 |
|  | 0.45 | 0.452 |
| NS | 0.15 | 0.178 |
|  | 0.30 | 0.323 |
|  | 0.45 | 0.486 |

^1^ *Values based on triplicate determinations*.

*SS, sodium selenite; SY, selenium yeast; SM, selenomethionine; SO, hydroxyl-selenomethionine; NS, nano-selenium*.

***Supplementary TABLE S3*** *Primer sequences for real-time PCR amplification*

| **Gene** | **GenBank identity** | **Primer sequences** |
| --- | --- | --- |
| β-actin | NM_205518.1 | Forward:5′-ACCTGAGCGCAAGTACTCTGTCT-3′ Reverse :5′-CATCGTACTCCTGCTTGCTGAT-3′ |
| *GAPDH* | NM_204305.1 | Forward:5′-CTTTGGCATTGTGGAGGGTC-3′ Reverse :5′-ACGCTGGGATGTGTTCTGG-3′ |
| *Gpx1* | NM_001277853.2 | Forward:5′-ACGGCGCATCTTCCAAAG-3′ Reverse :5′-TGTTCCCCCAACCATTTCTC-3′ |
| *Gpx4* | NM_001346448.1 | Forward:5′-CTTCGTCTGCATCATCACCAA-3′ Reverse :5′-TCGACGAGCTGAGTGTAATTCAC-3′ |
| *Dio1* | NM_001097614.1 | Forward:5′-GCGCTATACCACAGGCAGTA-3′ Reverse :5′-GGTCTTGCAAATGTCACCAC-3′ |
| *Selenop* | NM_001031609.2 | Forward:5′-CCAAGTGGTCAGCATTCACATC-3′ Reverse :5′-ATGACGACCACCCTCACGAT-3′ |
| *Selenou* | NM_001193519.2 | Forward:5′-GATGCTTTCAGGCTTCTTCC-3′ Reverse :5′-CTGTCTTCCTGCTCCAATCA-3′ |

*Gpx1, glutathione peroxidase 1; Gpx4, glutathione peroxidase 4; Dio1, iodothyronine deiodinase 1;* *Selenop, selenoprotein P; Selenou, Selenoprotein U*.
